# Comparison of Clinical Outcomes and Survival Rates Between Short and Standard Dental Implants in the Posterior Jaw: A Retrospective Cohort Study

**DOI:** 10.7759/cureus.112307

**Published:** 2026-07-08

**Authors:** Md Siraj ur Rahman, Ashank Mishra, Sree Ram Subba Reddy Gudimetla, Kamlesh Singh, Karandeep Singh, Jay Taank

**Affiliations:** 1 Department of Prosthodontics, Government Dental College and Hospital, Hyderabad, IND; 2 Department of Periodontics, Government Dental College and Hospital, Hyderabad, IND; 3 Department of Oral and Maxillofacial Surgery, Priyadarshini Dental College and Hospital, Pandur, IND; 4 Department of Periodontology, Swargiya Dadasaheb Kalmegh Smruti Dental College and Hospital, Nagpur, IND; 5 Department of Oral and Maxillofacial Surgery, Faculty of Dental Sciences, Shree Guru Gobind Singh Tricentenary University, Gurugram, IND; 6 Department of Oral and Maxillofacial Surgery, Chhattisgarh Dental College and Research Institute, Rajnandgaon, IND

**Keywords:** dental implant failure, dental implants, implant survival, posterior jaw, retrospective studies

## Abstract

Introduction: Short dental implants have been proposed as a minimally invasive alternative to standard implants for rehabilitation of posterior edentulous regions with limited bone availability. However, concerns remain regarding their long-term survival and risk of failure compared to standard-length implants. This study aimed to compare the survival and failure rates of short (<10 mm) and standard (≥10 mm) dental implants placed in the posterior maxilla and mandible.

Materials and Methods: This retrospective cohort study included 207 patients who received 363 dental implants in the posterior jaw. The implants were categorized as short implants (<10 mm; n = 141) and standard implants (≥10 mm; n = 222). Demographic characteristics, implant-related variables, and clinical outcomes were retrieved from the patient records. Implant failure, survival rates, and the influence of jaw location on implant outcomes were assessed. Statistical analyses included chi-square test or Fisher's exact test, independent-samples t-tests, and odds ratio calculations. Statistical significance was set at p < 0.05.

Results: The mean age of the study population was 52.1 ± 10.5 years, with 142 (68.6%) males and 65 (31.4%) females. Overall, implant failure was significantly higher among short implants than among standard implants (p < 0.001). Standard implants demonstrated significantly lower odds of failure than short implants (OR = 0.29; 95% CI: 0.15-0.57). In the maxilla, the failure rates were 18 (28.6%) for short implants and 8 (8.5%) for standard implants (p = 0.002), whereas in the mandible, the corresponding rates were 10 (12.8%) and 7 (5.5%), respectively (p = 0.110). The cumulative survival rates were lower for short implants as compared to standard implants.

Conclusion: Standard dental implants demonstrated significantly higher survival rates and lower failure rates than short implants in posterior jaw rehabilitation. Although short implants remain a useful treatment option in anatomically restricted sites, careful case selection is necessary, particularly in the posterior maxilla, where the risk of failure appears to be greater.

## Introduction

Dental implants are widely recognized as a predictable and effective treatment option for the rehabilitation of partially or completely edentulous patients. Successful implant therapy depends on several factors, including adequate bone volume, primary stability, implant design, and favorable biomechanical loading [1]. However, implant placement in the posterior regions of the jaws frequently presents clinical challenges because of reduced alveolar bone height resulting from post-extraction resorption, maxillary sinus pneumatization, and proximity to anatomical structures such as the inferior alveolar nerve [2,3]. Conventional management of these situations often requires advanced surgical procedures, including bone grafting, sinus floor elevation, or nerve transposition, which may increase treatment complexity, cost, and patient morbidity [3,4].

Short dental implants have been introduced as a less invasive alternative to augmentation procedures, offering the possibility of implant-supported rehabilitation in areas with limited bone availability [5]. Improvements in implant surface characteristics, macrodesign, and surgical protocols have enhanced the clinical performance of short implants and expanded their use. Nevertheless, concerns remain regarding their long-term survival and mechanical behavior because of their reduced implant length and potentially higher biomechanical stress concentrations [6,7]. While several studies have investigated the outcomes of short implants, the available evidence remains inconsistent, particularly with respect to implant survival and failure rates in the posterior maxilla and mandible [6-8].

Standard dental implants are considered the treatment of choice when sufficient alveolar bone height and width are available, as they provide greater bone-to-implant contact, favorable load distribution, and excellent long-term survival. However, in patients with limited vertical bone height, particularly in the posterior maxilla because of maxillary sinus pneumatization or in the posterior mandible because of the proximity of the inferior alveolar nerve, placement of standard implants may require additional augmentation procedures such as sinus floor elevation or vertical ridge augmentation. In such situations, short dental implants have emerged as a minimally invasive alternative, reducing surgical morbidity, treatment time, cost, and patient discomfort while avoiding complex reconstructive procedures. Nevertheless, careful case selection is essential, as short implants may be associated with greater biomechanical stress and a higher risk of failure, particularly in areas with poor bone quality or high occlusal loading. Therefore, the choice between short and standard implants should be individualized based on the available bone volume, anatomical limitations, bone quality, occlusal considerations, and patient-related factors [4-8].

Therefore, the present retrospective cohort study was conducted to compare the clinical outcomes of short (<10 mm) and standard (≥10 mm) dental implants placed in the posterior jaws. The primary objective was to compare the survival and failure rates of short and standard implants. The secondary objectives were to assess the influence of implant length on the risk of implant failure, compare failure patterns between the maxilla and mandible, evaluate differences in implant-related characteristics such as prosthetic retention, implant diameter, and follow-up duration, and analyze cumulative implant survival over time.

## Materials and methods

This retrospective cohort study was conducted using the records of patients who underwent dental implant placement in the posterior regions of the maxilla and mandible at the Department of Oral and Maxillofacial Surgery, Priyadarshini Dental College and Hospital, Pandur, India, between January 2014 and December 2024. Patient records with a follow-up period ranging from 12 to 36 months were reviewed. Ethical approval for the study was obtained from the Institutional Ethics Committee (Approval No. PDCH/SAP/03/2025/34; dated March 24, 2025) prior to data collection, and all procedures were conducted in accordance with the principles of the Declaration of Helsinki.

Sample size estimation

A post-hoc sample size calculation was performed using G*Power (Heinrich Heine University, Düsseldorf, Germany) to determine whether the available sample was adequate for detecting differences in implant failure rates between the two implant-length groups. Based on observed failure proportions of 19.9% for short implants and 6.8% for standard implants, an effect size (h) of 0.39 was calculated using arcsine transformation [9]. Considering a two-sided alpha level of 0.05 and a statistical power of 80%, a minimum sample of 302 implants was required. The final study sample comprised 363 implants, indicating adequate statistical power for the primary outcome assessment.

Study population

The records of 207 patients who received 363 implants in the posterior maxilla and mandible were screened for eligibility. Patients aged 18 years or older who had undergone implant-supported rehabilitation in posterior edentulous regions and had complete clinical and radiographic records with a minimum follow-up period of 12 months were included. Patients with incomplete documentation, inadequate follow-up records, untreated periodontal disease at the time of implant placement, active oral infections, or missing outcome data were excluded. Demographic variables, including age, sex, smoking status, and the presence of diabetes mellitus, were extracted from the patient records. Implant-related variables, including implant length, implant diameter, jaw location, prosthetic retention type, follow-up duration, and implant survival status, were also recorded.

Implant placement procedure

All implant surgeries were performed by experienced implant surgeons (MSR and AM) following standardized surgical protocols. Preoperative assessment included a detailed clinical examination and radiographic evaluation to determine bone availability, assess anatomical limitations, and formulate an appropriate treatment plan. Implant length selection was based on the available bone height and prosthetic requirements. Implants measuring less than 10 mm in length were categorized as short implants, whereas implants measuring ≥10 mm were classified as standard implants [9].

All patients received implants from the Osstem Implant System (Osstem Implant Co., Ltd., Seoul, South Korea). Surgical procedures were performed under local anesthesia using a conventional crestal incision, followed by elevation of a full-thickness mucoperiosteal flap. Osteotomy preparation was carried out using sequential drills according to the manufacturer's recommended drilling protocol under copious sterile saline irrigation to minimize thermal injury to the surrounding bone. Implant fixtures were inserted into the prepared osteotomy sites until adequate primary stability was achieved. Following implant placement, cover screws or healing abutments were attached according to the surgical protocol, and the surgical sites were closed using interrupted sutures.

Postoperatively, patients were prescribed analgesics and antibiotics when indicated and were provided with standardized instructions regarding oral hygiene and dietary precautions. Sutures were removed following satisfactory soft tissue healing. After an appropriate healing period to allow osseointegration, prosthetic rehabilitation was completed using either screw-retained or cement-retained restorations according to the prosthetic treatment plan. All patients were subsequently enrolled in a maintenance program involving periodic clinical and radiographic evaluations throughout the follow-up period.

Prosthetic rehabilitation

After the completion of the healing period and confirmation of satisfactory osseointegration, prosthetic rehabilitation was initiated. Depending on clinical requirements, restorations were fabricated as either screw-retained or cement-retained prostheses. Occlusal adjustments were performed to ensure even force distribution and minimize excessive loading on the implants. The patients were enrolled in a maintenance program involving periodic clinical and radiographic evaluations.

Outcome assessment

The primary outcome measure was implant failure. Implant failure was defined as implant mobility, loss of osseointegration, persistent peri-implant infection requiring implant removal, implant fracture, or removal of the implant for any biological or mechanical reason during the follow-up period [10]. Implant survival was defined as the continued presence of a clinically stable and functional implant throughout the observation period. Survival rates were calculated by subtracting the number of failed implants from the total number of implants in each study group and expressing the result as a percentage [11]. Secondary outcome measures included the association between implant length and the risk of implant failure, the distribution of failures according to jaw location, and cumulative implant survival over time. Failure rates were evaluated separately for implants placed in the maxilla and mandible.

Reliability and data quality control

Data extraction was performed independently by two calibrated investigators (KS and JT). Prior to data collection, examiner calibration was conducted using a subset of 20 patient records that were not included in the final analysis. Any discrepancies in data extraction were resolved through discussion and consensus. Inter-examiner agreement was assessed using Cohen's kappa coefficient for categorical variables and the intraclass correlation coefficient (ICC) for continuous variables. The kappa coefficient was 0.88, indicating excellent agreement. The ICC values were 0.91 for implant diameter measurements and 0.94 for follow-up duration measurements, demonstrating excellent inter-examiner reliability. Kappa and ICC values greater than 0.80 were considered indicative of excellent reliability.

Statistical analysis

Data were entered into a spreadsheet and analyzed by a statistician (SRSRG) using IBM SPSS Statistics for Windows, Version 29 (Released 2022; IBM Corp., Armonk, New York). Continuous variables were assessed for normality using the Shapiro-Wilk test. Normally distributed data are presented as mean ± standard deviation and were compared using independent-samples t-tests. Categorical variables are presented as frequencies and percentages and were compared using the chi-square test or Fisher's exact test when the expected cell count was less than five. Yates' continuity correction was applied to 2 × 2 contingency tables for the analysis of implant failure. Odds ratios (ORs) with 95% confidence intervals (CIs) were calculated to quantify the association between implant length and implant failure. Statistical significance was established at p < 0.05.

## Results

A total of 207 patients who received 363 dental implants in the posterior jaw were included in the study. The mean age of the study population was 52.1 ± 10.5 years. Of the 207 patients, 142 (68.6%) were males and 65 (31.4%) were females. Smoking was reported in 58 patients (28.0%), whereas diabetes mellitus was present in 45 patients (21.7%) (Table 1).

**Table 1:** Demographic characteristics of the study population. Data are presented as mean ± SD or n (%) where n denotes the number of patients. SD: standard deviation.

Parameters		Total n = 207
Age (years) and Mean SD		52.1 ± 10.5
Sex, n (%)	Male	142 (68.6)
	Female	65 (31.4)
Smoking, n (%)	Yes	58 (28.0)
	No	149 (72.0)
Diabetes, n (%)	Yes	45 (21.7)
	No	162 (78.3)

Of the 363 implants evaluated, 141 (38.8%) were short implants (<10 mm) and 222 (61.2%) were standard implants (≥10 mm). No statistically significant differences were observed between the two groups in jaw distribution (p = 0.66), prosthetic retention type (p = 0.78), implant diameter (p = 0.61), or follow-up duration (p = 0.41), indicating baseline comparability (Table 2).

**Table 2:** Comparison of implant characteristics between short and standard implants. Data are presented as mean ± SD or n (%); p < 0.05 was considered statistically significant. SD: standard deviation; χ²: chi-square test; t: independent t-test.

Characteristic		Total (N = 363)	Short implants <10 mm (n = 141, 38.8%)	Standard implants ≥10 mm (n = 222, 61.2%)	Test value	p-value
Jaw, n (%)	Maxilla	157 (43.3)	63 (44.7)	94 (42.3)	χ² = 0.19	0.66
	Mandible	206 (56.7)	78 (55.3)	128 (57.7)		
Prosthetic retention, n (%)	Screw‑retained	260 (71.6)	100 (70.9)	160 (72.1)	χ² = 0.08	0.78
	Cement‑retained	103 (28.4)	41 (29.1)	62 (27.9)		
Diameter (mm)	Mean ± SD	4.05 ± 0.40	4.10 ± 0.51	4.02 ± 0.48	t = 0.51	0.61
Follow‑up (months)	Mean ± SD	25.30 ± 7.61	24.80 ± 7.89	25.60 ± 7.40	t = 0.82	0.41

Implant failure analysis demonstrated significantly higher failure rates among short implants than among standard implants. Overall, 28 (19.9%) short implants failed, whereas only 15 (6.8%) standard implants failed, representing a significant difference (p < 0.001). Standard implants demonstrated significantly lower odds of failure than short implants (OR = 0.29; 95% CI: 0.15-0.57) (Table 3).

**Table 3:** Comparison of implant failure rates between short and standard implants according to implant location. Data are presented as n (%); chi-square test with Yates' continuity correction was used for comparisons; odds ratios are presented with 95% confidence intervals; *p < 0.05 was considered statistically significant. OR: odds ratio; CI: confidence interval.

Location	Short (<10 mm)		Standard (≥10 mm)		χ²	p-value	OR	95% CI
	n	Failures, n (%)	n	Failures, n (%)				
Maxilla	63	18 (28.6%)	94	8 (8.5%)	9.582	0.002*	0.23	0.09-0.58
Mandible	78	10 (12.8%)	128	7 (5.5%)	2.557	0.110	0.39	0.14-1.08
Overall	141	28 (19.9%)	222	15 (6.8%)	12.947	< 0.001*	0.29	0.15-0.57

**Figure 1:**
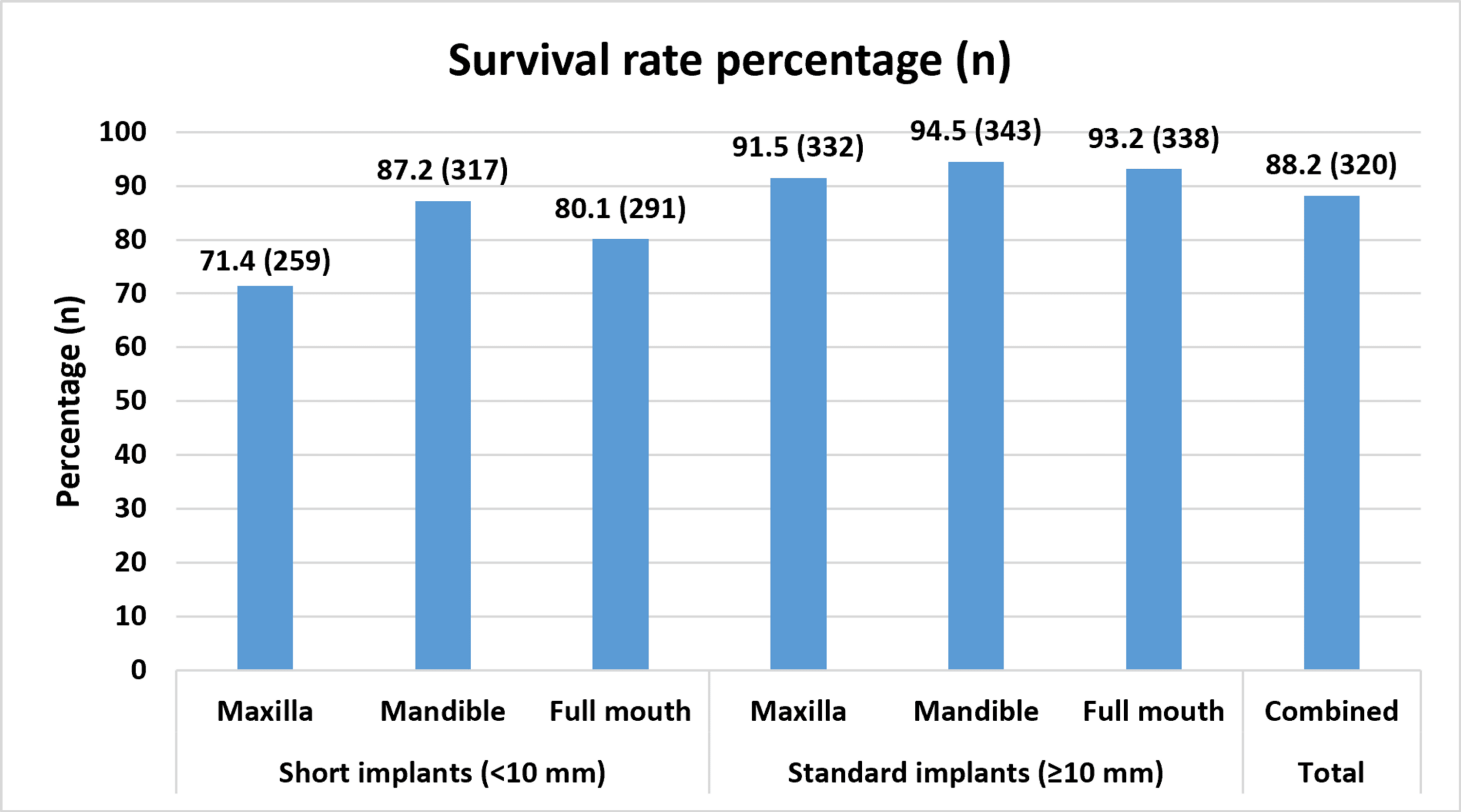
Survival analysis shows percentage of implant success in both study groups. Data shown as percentage (frequency).

When analyzed according to jaw location, short implants placed in the maxilla exhibited a significantly higher failure rate than standard implants (p = 0.002). In the mandible, failure rates were also higher for short implants than for standard implants; however, this difference was not statistically significant (p = 0.110) (Table 3). The cumulative survival rates were lower for short implants as compared to standard implants (Figure 1).

## Discussion

This retrospective cohort study compared the clinical performance of short (<10 mm) and standard (≥10 mm) dental implants placed in the posterior regions of the jaws. The findings demonstrated significantly higher failure rates and lower cumulative survival rates among short implants than among standard implants. Although both implant types achieved acceptable clinical outcomes, implant length appeared to have a substantial influence on long-term survival, particularly in the posterior maxilla.

The overall implant failure rate observed in this study was significantly higher for short implants than for standard implants. Correspondingly, the cumulative survival rate was lower for short implants than for standard implants. These findings suggest that reduced implant length may compromise long-term biomechanical stability and resistance to functional loading. Short implants inherently provide a smaller bone-implant contact area, which may increase stress concentrations at the crestal bone level and potentially predispose the implant to biological or mechanical complications [6,7]. The significantly greater risk of failure observed among short implants further supports the influence of implant length on treatment outcomes.

Early studies suggested that shorter implants were associated with increased failure rates because of reduced osseointegration surface area and less favorable load distribution. Higher crown-to-implant ratios frequently associated with short implants may further amplify occlusal forces and contribute to peri-implant bone stress [12]. These biomechanical considerations may partially explain the greater number of failures observed among short implants in the present study.

In contrast, Monje et al. [9] conducted a meta-analysis of 13 prospective clinical trials involving 1,955 implants and found no significant difference in long-term survival between short (<10 mm) and standard (≥10 mm) implants, with estimated survival rates of 88.1% and 86.7%, respectively. Advances in implant macrogeometry, thread design, and surface modification have substantially improved the clinical predictability of short implants. Nevertheless, many of these studies involved carefully selected patients, controlled loading protocols, and highly standardized treatment conditions. The present study reflects outcomes observed in routine clinical practice and may therefore provide a more realistic assessment of implant performance under everyday treatment conditions.

Pommer et al. [13] reported that short implants represent a valuable treatment option for patients with reduced alveolar bone height and may reduce the need for invasive bone augmentation procedures. Although short implants offer important advantages in anatomically compromised sites, the present study observed significantly higher failure rates than standard implants, highlighting the need for careful patient selection.

An important finding of this study was the influence of jaw location on implant survival. In the maxilla, short implants exhibited a significantly higher failure rate than standard implants; however, the difference in the mandible did not reach statistical significance. This observation may be attributed to differences in bone quality between the two jaws. The posterior maxilla is frequently characterized by low-density trabecular bone and reduced cortical support, which may compromise primary stability and osseointegration. Conversely, the posterior mandible generally possesses denser cortical bone that provides improved mechanical anchorage and may compensate for reduced implant length [14,15].

The findings of the present study are further supported by the systematic review summarized by Carr [16], which demonstrated that the survival of short implants improves with increasing implant length and is generally higher in the mandible than in the maxilla. The review also reported lower failure rates among non-smokers than among smokers, highlighting the influence of patient-related factors on implant outcomes. Similarly, the present study observed significantly higher failure rates for short implants, particularly in the maxilla, whereas the difference in the mandible was not statistically significant. These findings emphasize the importance of anatomical location and patient selection in determining the success of short implants.

Queiroz et al. [17] evaluated short implants placed in the posterior mandible and observed a significantly lower survival rate for short implants (87.5%) than for regular implants (100%) after 90 days of follow-up. The authors concluded that although short implants may exhibit reduced survival, they remain a reasonable alternative for the rehabilitation of severely resorbed jaws, where bone augmentation procedures can be avoided. Similarly, the present study demonstrated significantly higher failure rates and lower survival rates among short implants, supporting the need for careful case selection when using short implants in anatomically compromised sites. Comparable findings have been reported in previous studies [18,19].

The comparable distribution of implant location, prosthetic retention type, implant diameter, and follow-up duration between the study groups strengthened the validity of the findings. The absence of significant differences in the measured baseline variables suggests that the observed variation in implant survival may be related, at least in part, to implant length. Furthermore, Kaplan-Meier survival analysis demonstrated consistently lower survival probabilities for short implants throughout the follow-up period, supporting the robustness of the primary outcome.

From a clinical perspective, the results suggest that standard implants remain the preferred treatment option when adequate bone volume is available. However, short implants may still serve as a valuable alternative in situations where anatomical limitations preclude the placement of longer implants or when patients are unwilling or unsuitable to undergo augmentation procedures. Careful patient selection, meticulous surgical technique, achievement of optimal primary stability, and strict control of occlusal loading are essential when short implants are considered. Particular caution should be exercised when placing short implants in the posterior maxilla because of the higher risk of failure in this region.

The present study had several limitations that should be acknowledged. The retrospective design limited control over potential confounding variables and introduced the possibility of selection bias, particularly because short implants are often placed in anatomically challenging sites. Several clinically relevant variables, including implant surface characteristics, insertion torque values, bone quality, grafting procedures, loading protocols, prosthetic design, occlusal factors, and parafunctional habits, were not consistently available in the clinical records and therefore could not be included in the analysis. In addition, the statistical analyses were primarily univariate and did not adjust for potential confounders. Furthermore, some patients received more than one implant, resulting in implant-level observations that may not have been completely independent. The follow-up period also varied among patients, and outcomes beyond 36 months could not be evaluated. Consequently, the findings should be interpreted with appropriate caution and viewed as associations observed within this retrospective cohort rather than evidence of a causal relationship. Despite these limitations, the study included a relatively large implant sample, demonstrated adequate statistical power, and provides clinically relevant information regarding the comparative survival of short and standard implants in posterior jaw rehabilitation.

## Conclusions

Within the limitations of this retrospective cohort study, standard dental implants were associated with higher survival rates and lower failure rates than short implants in the posterior regions of the jaws. This difference was more pronounced in the posterior maxilla, where higher failure rates were observed among short implants. Although short implants remain a valuable treatment option in patients with limited bone availability or anatomical constraints, careful case selection and treatment planning are essential. Given the retrospective design and the potential influence of unmeasured confounding factors, these findings should be interpreted with caution. Further well-designed prospective studies with adjustment for relevant clinical variables are needed to confirm these observations.

## References

[1] Shahapur SG, Patil K, Manhas S, Datta N, Jadhav P, Gupta S (2024). Predictive factors of dental implant failure: a retrospective study using decision tree regression. Cureus.

[2] Agarwal MD, Dodamani G, Dodamani A, Nagaral S, Ronad S, Pungle A, Gupta S (2025). Implant stability in narrow posterior alveolar ridges: a randomized controlled trial comparing conventional ridge split and osseodensification. Cureus.

[3] Lam L, Ivanovski S, Lee RS (2024). Alveolar ridge preservation in posterior maxillary teeth for reduction in the potential need for sinus floor elevation procedures: a pilot study. Clin Oral Implants Res.

[4] Mahardawi B, Jiaranuchart S, Arunjaroensuk S, Dhanesuan K, Mattheos N, Pimkhaokham A (2025). The clinical benefit of alveolar ridge preservation in the posterior maxilla: a systematic review and meta-analysis. Sci Rep.

[5] Jain N, Gulati M, Garg M, Pathak C (2016). Short implants: new horizon in implant dentistry. J Clin Diagn Res.

[6] Wyatt CC, Zarb GA (1998). Treatment outcomes of patients with implant-supported fixed partial prostheses. Int J Oral Maxillofac Implants.

[7] Bahat O (2000). Brånemark system implants in the posterior maxilla: clinical study of 660 implants followed for 5 to 12 years. Int J Oral Maxillofac Implants.

[8] Rameh S, Menhall A, Younes R (2020). Key factors influencing short implant success. Oral Maxillofac Surg.

[9] Monje A, Chan HL, Fu JH, Suarez F, Galindo-Moreno P, Wang HL (2013). Are short dental implants (&lt;10 mm) effective? A meta-analysis on prospective clinical trials. J Periodontol.

[10] Misch CE, Perel ML, Wang HL (2008). Implant success, survival, and failure: the International Congress of Oral Implantologists (ICOI) Pisa Consensus Conference. Implant Dent.

[11] Albrektsson T, Zarb G, Worthington P, Eriksson AR (1986). The long-term efficacy of currently used dental implants: a review and proposed criteria of success. Int J Oral Maxillofac Implants.

[12] Yuan X, Liu Y, Yang Y, Ren M, Luo L, Zheng L, Liu Y (2023). Effect of short implant crown-to-implant ratio on stress distribution in anisotropic bone with different osseointegration rates. BMC Oral Health.

[13] Pommer B, Frantal S, Willer J, Posch M, Watzek G, Tepper G (2011). Impact of dental implant length on early failure rates: a meta-analysis of observational studies. J Clin Periodontol.

[14] Olmedo-Gaya MV, Romero-Olid MN, Ocaña-Peinado FM, Vallecillo-Rivas M, Vallecillo C, Reyes-Botella C (2023). Influence of different surgical techniques on primary implant stability in the posterior maxilla: a randomized controlled clinical trial. Clin Oral Investig.

[15] Liu C, Xing Y, Li Y, Lin Y, Xu J, Wu D (2022). Bone quality effect on short implants in the edentulous mandible: a finite element study. BMC Oral Health.

[16] Carr AB (2012). Survival of short implants is improved with greater implant length, placement in the mandible compared with the maxilla, and in nonsmokers. J Evid Based Dent Pract.

[17] Queiroz TP, Aguiar SC, Margonar R, de Souza Faloni AP, Gruber R, Luvizuto ER (2015). Clinical study on survival rate of short implants placed in the posterior mandibular region: resonance frequency analysis. Clin Oral Implants Res.

[18] Renouard F, Nisand D (2006). Impact of implant length and diameter on survival rates. Clin Oral Implants Res.

[19] Abdel-Halim M, Issa D, Chrcanovic BR (2021). The impact of dental implant length on failure rates: a systematic review and meta-analysis. Materials (Basel).

